# Quality by Design Based Formulation Study of Meloxicam-Loaded Polymeric Micelles for Intranasal Administration

**DOI:** 10.3390/pharmaceutics12080697

**Published:** 2020-07-24

**Authors:** Bence Sipos, Piroska Szabó-Révész, Ildikó Csóka, Edina Pallagi, Dorina Gabriella Dobó, Péter Bélteky, Zoltán Kónya, Ágota Deák, László Janovák, Gábor Katona

**Affiliations:** 1 Faculty of Pharmacy, Institute of Pharmaceutical Technology and Regulatory Affairs, University of Szeged, H-6720 Szeged, Hungary; sipos.bence96@gmail.com (B.S.); revesz@pharm.u-szeged.hu (P.S.-R.); csoka@pharm.u-szeged.hu (I.C.); edina.pallagi@pharm.u-szeged.hu (E.P.); dobo.dorina@pharm.u-szeged.hu (D.G.D.); 2 Faculty of Science and Informatics, Department of Applied & Environmental Chemistry, H-6720 Szeged, Hungary; beltekyp@chem.u-szeged.hu (P.B.); konya@chem.u-szeged.hu (Z.K.); 3 Interdisciplinary Excellence Centre, Department of Physical Chemistry and Materials Science, H-6720 Szeged, Hungary; deak.agota@stud.u-szeged.hu (Á.D.); janovakl@chem.u-szeged.hu (L.J.)

**Keywords:** NSAID, nanoDDS, polymeric micelle, quality by design, preformulation study, freeze-drying, reconstituted nasal formulation, nose-to-brain delivery, solubility enhancement, prediction of IVIVC

## Abstract

Our study aimed to develop an “ex tempore” reconstitutable, viscosity enhancer- and preservative-free meloxicam (MEL)-loaded polymeric micelle formulation, via Quality by Design (QbD) approach, exploiting the nose-to-brain pathway, as a suitable tool in the treatment of neuroinflammation. The anti-neuroinflammatory effect of nose-to-brain NSAID polymeric micelles was not studied previously, therefore its investigation is promising. Critical product parameters, encapsulation efficiency (89.4%), Z-average (101.22 ± 2.8 nm) and polydispersity index (0.149 ± 0.7) and zeta potential (−25.2 ± 0.4 mV) met the requirements of the intranasal drug delivery system (nanoDDS) and the targeted profile liquid formulation was transformed into a solid preservative-free product by freeze-drying. The viscosity (32.5 ± 0.28 mPas) and hypotonic osmolality (240 mOsmol/L) of the reconstituted formulation provides proper and enhanced absorption and probably guarantees the administration of the liquid dosage form (nasal drop and spray). The developed formulation resulted in more than 20 times faster MEL dissolution rate and five-fold higher nasal permeability compared to starting MEL. The prediction of IVIVC confirmed the great potential for in vivo brain distribution of MEL. The nose-to-brain delivery of NSAIDs such as MEL by means of nanoDDS as polymeric micelles offers an innovative opportunity to treat neuroinflammation more effectively.

## 1. Introduction

Among the leading mortality causes, neurodegenerative diseases are in the forefront. Their increasing prevalence poses a challenge to find the effective therapy worldwide. Alzheimer’s disease (AD) is one of the most common causes of dementia, accounting for approximately 60–70%. The estimated proportion of the general population aged 60 and over with dementia at a given time is between 5–8%. The total number of people with dementia is projected to reach 82 million in 2030 and 152 in 2050 because with the increasing life expectancy, a dramatic rise in the number of age-associated diseases can be predicted, therefore there is an increased demand for easy-to-apply medications which can improve patient adherence [1]. Strategies for facilitating medication adherence in patients with dementia include applying as few medicines as possible, tailoring dose regimens to personal habits and minimizing the drug dosing intervals as much as possible [2], therefore smart drug delivery systems are required. Within symptomatic therapies, the treatment of cyclooxygenase (COX) enzymes mediated neuroinflammation plays a prominent role, which is primarily attributable to nonsteroidal anti-inflammatory drugs (NSAIDs). Inhibition of COX enzymes can reduce amyloid deposition and inhibit glia activity, which has been already demonstrated in a mouse model [3,4]. Subsequently, numerous studies have demonstrated their pharmacological activity in the central nervous system (CNS). Treating neuroinflammation by NSAIDs could be possibly based on protection by depolarizing the mitochondria and inhibiting the calcium uptake, due to the ionizable carboxylic group, having a similar effect to mild mitochondrial uncouplers [5,6]. However, the blood–brain barrier (BBB) is an obstacle to the therapy because it prevents the release of active substances that could be used by its protective function [7]. In addition to the appropriate technological formulation, the route of administration also plays a pivotal role [8].

The intranasal administration route can be exploited both in systemic and local therapy. Due to the high surface area and rich vascularization of nasal mucosa, drugs or drug-delivery systems can be easily absorbed from the nasal cavity. This direct way can also protect the API from the first-pass metabolism, thus preserving its pharmacologically activity [9,10,11]. Studies have shown that AD starts in the entorhinal cortex, which is associated with olfactory nerves and begins to spread in a corresponding pattern [12], therefore the intranasal administration of the API can be advantageous. The potential for colloidal carriers to increase drug bioavailability is in the focus of research [13]. Nano drug delivery systems (nanoDDSs) are able to bypass the BBB through the trigeminal and olfactory nerves, resulting in higher brain concentration of the incorporated active pharmaceutical ingredient, moreover their application is required in the case of NSAID administration, because of their low solubility at the pH (5.3–5.6) of the nasal cavity and low residence time (10–15 min) due to mucociliary clearance [14].

Our research group has been working on nanoDDSs aiming to transfer APIs through or bypassing the blood–brain barrier exploiting the applicability of NSAIDs in a nasal formulation. Based on the previous results of the research group, a good in vitro–in vivo correlation (IVIVC) was achieved by focusing on the administered API’s concentration in the brain. Different nanotechnological methods were applied in order to achieve this goal. As a disintegration method, the co-grinding process with different excipients was used, which resulted in nanonization of the drug, thereby offering higher permeability to biologic barriers and therefore transporting them across the nasal mucosa [15,16]. Besides the top–down methods, bottom–up techniques were also successfully applied, in order to incorporate the API into nanoDDS. Liposomes as lipid-based nanoDDS with reduced particle size and the bilayer lipid membrane provided high and fast transport of encapsulated lamotrigine through the nasal mucosa [17]. The in vivo studies of meloxicam (MEL)-containing human serum albumin nanoparticles corroborated the in vitro dissolution and permeability studies contributed to determine the criteria of trans-epithelial and axonal transport ensuring higher cerebral concentration of the API [18]. These results gave the background of the investigation of polymeric micelles, which may offer more favorable pharmacokinetics and higher protection to the API.

Polymeric micelles are composed of amphiphilic graft copolymers, consisting of a hydrophilic and a hydrophobic block moiety. They show concentration dependent variation in the physicochemical properties similar to classic surfactant molecules, such as spontaneously self-assembling above the critical micellar concentration (CMC) or forming association colloids into nanosized micelles above the critical micellar temperature. Among their benefits, they can incorporate drugs with poor water solubility and permeability into their hydrophobic core while the hydrophilic shell is responsible for solubilizing. This property predicts increased bioavailability and offers the possibility to administer the drug via alternative administration routes [19,20,21]. Two main ways of drug release are known from the micellar core: one is the dissociation of the micelle breaking down to monomers and the other is based on drug–polymer bond breakage in the micellar core allowing diffusion from there. Simple diffusion from the core can be controlled by different stimuli (e.g., pH) with appropriate engineering [22].

Along with the development of novel drug delivery conventional and novel synthetic or natural excipients provide opportunities to design dosage forms with the required features including their bioavailability [23]. In the composition design of polymeric micelles amphiphilic micelle forming graft copolymer Soluplus^®^ was chosen, due to its advantageous properties: low CMC value in water, mucoadhesion, biodegradability. Soluplus^®^ has low toxicity, according to the manufacturer data (BASF SE, Ludwigshafen, Germany), the oral and dermal median lethal dose values (lethal dose, 50%; LD50) were both >5 g/kg, therefore its nasal application is safe. Its amphiphilic nature is due to the following composition: 13% polyethylene glycol (PEG) 6000 as hydrophilic, 57% vinyl caprolactam and 30% vinyl acetate as the hydrophobic block. The PEG chain forms the strain on which the other two lipophilic monomer side chains are incorporated. Due to its bifunctional character, it can be used for preparing solid solutions as well as for solubilizing active substances with poor water solubility as many previous research studies have shown [24,25].

This study reports MEL as a model drug, which could be a promising active substance for the treatment of neuroinflammation with selective COX-2 inhibition. With this selectivity we can exclude the opportunity to harm the gastric mucosa. It has poor water solubility (BCS class II), especially in the pH range of the nasal cavity, which impedes liberation from the formulation, resulting in low absorption as well as bioavailability [26]. By encapsulating MEL in a polymeric micelle, the drug could be absorbed by the nasal epithelial cells, thereby delivering it directly through axonal transport pathway into the CNS [18,27].

Quality by design (QbD) is a systematic industrial approach to the formulation of pharmaceutical products, which begins with predefined objectives and emphasizes product and process understanding and process control, based on quality risk management [28,29]. In addition, submissions for marketing authorization must contain QbD elements according to the regulatory authorities’ requirements. Based on ICH guidelines (Q8 R2, Q9, Q10, Q11) the establishment and the control of quality must be grounded on risk-based concepts and principles. The essential elements in a QbD approach are the following: determining the quality target product profile (QTPP), selecting the critical quality attributes (CQAs) and critical process parameters (CPPs), risk assessment (RA), design of experiments (DoE), developing a design space (DS) with a proper control strategy and eventually managing the product’s lifecycle, including the aspects of continuous improvement. The CQAs are related to the quality, safety and efficacy profile of the product while the critical material attributes (CMAs) and the CPPs are related to the selected production method. With evaluating a risk estimation matrix, we can truly generate a risk-and-knowledge-based quality management method by ranking CQAs and CPPs regarding their degree of impact on the targeted product quality [30]. This classical QbD model was also favorable in terms of preformulation studies of nanoDDSs, as well as for nasal formulations [31,32,33].

Our study aimed to formulate “ex tempore” reconstitutable MEL-loaded Soluplus^®^ polymeric micelles for intranasal application, exploiting the nose-to-brain pathway optimized via quality by design (QbD) approach. Preformulation studies were carried out to develop a stable nasal formulation that is preservative-free and does not contain added viscosity enhancer. The experimental design and in vitro characterization were focused on the IVIVC (in vitro-in vivo correlation) as guidance to reduce the number of animal studies [34,35,36] which was predicted using a mathematical model.

## 2. Materials and Methods

### 2.1. Materials

MEL (4-hydroxy-2-methyl-*n*-(5-methyl-2-thiazolyl)-2 H-benzothiazine-3-carboxamide-1,1-dioxide) was applied as model drug and acquired from EGIS Plc. (Budapest, Hungary). Soluplus^®^ (BASF GmbH, Hanover, Germany) was used as micelle-forming agent. Ethanol (Merck, Ltd., Budapest, Hungary) as organic solvent was used during our experiment. Microcrystalline sodium hydroxide (NaOH) as formulation excipient, chemicals for Simulated Nasal Electrolyte Solution (SNES) [16] which combined 8.77 g sodium chloride (NaCl), 2.98 g potassium chloride (KCl), 0.59 g and anhydrous calcium chloride (CaCl_2_) in 1000 mL of deionized water at pH 5.6 as well as disodium phosphate (Na_2_HPO_4_), monopotassium phosphate (KH_2_PO_4_) for pH 7.4 Phosphate-buffered saline (PBS) dissolution media and the cryoprotectant d-trehalose dihydrate were acquired from Sigma-Aldrich Co., Ltd. (Budapest, Hungary). Purified water for the experiments was filtered using the Millipore Milli-Q^®^ (Merck, Ltd., Budapest, Hungary) 140 Gradient Water Purification System.

### 2.2. Initial Risk Assessment and Knowledge Space Development

The first step in QbD-based initial RA was the determination of the QTPP of the target product. The identification of CQAs and the CPPs of the formulation method was the second step. A primary knowledge space development was made as part of the QbD methodology and a cause and effect (Ishikawa) diagram was set up [37]. The LeanQbD^®^ software (QbD Works LLC, Fremont, CA, USA, www.qbdworks.com) was used for the RA procedure. First, the interdependence rating among QTPPs and CQAs and CPPs and CQAs was evaluated. A three-level scale was used to describe the relation between the parameters: “high” (H), “medium” (M) or “low” (L). Then a probability rating step was made, where a 0–10 scale was used for occurrence estimation of the CPPs where the values were assigned to a similar H/M/L ranking structure. As the output of the RA evaluation, Pareto diagrams [38] were generated presenting the numeric data and the ranking of the CQAs and CPPs according to their potential impact on product quality. Table 1 shows the defined requirements expressed as the QTPPs of MEL-containing polymeric micelles.

### 2.3. Production of MEL–Soluplus^®^ Polymeric Micelles via Box–Behnken Factorial Design Optimization

First, the appropriate amount of MEL was dissolved in ethanol under continuous stirring by dropwise addition of 1 M NaOH solution. The NaOH solution provided an alkaline medium, therefore the weak acidic character (pKa = 4.08) of MEL could easily deprotonate and dissolve in the organic solvent. Because of the oxo-enolic tautomeric balance a bright yellow solution was acquired. The next step was the dissolution of Soluplus^®^ in the solution of MEL under the constant stirring of the mixture for an hour. Then a Büchi R-210 (Büchi, Flawil, Switzerland) rotation vacuum evaporator was used to extract the solvents and a thin layer of matrix film was formed in the round-bottom flask. For the investigations, samples were collected into petri dishes then dried under vacuum for 6 h at ambient temperature (25 ± 2 °C) in a vacuum dryer to provide further solvent removal. The rehydration process was carried out with 6 mL purified water by mechanical agitation using a magnetic stirrer at 500 rpm. MEL’s concentration was designed to be constant (2.5 mg/mL) after the rehydration, which is a fraction of the active ingredient content of the currently marketed per os formulations (products contain 15 mg of MEL in their dosage form on the market). Then the rehydrated polymeric micelle formulation was freeze-dried into a stable product. Freeze-drying of solid products were carried out using ScanVac CoolSafe 100–9 (LaboGene, ApS, Lynge, Denmark) laboratory apparatus. Vials were filled with 1.5 mL of polymeric micelles solution and 5 *w/w*% of trehalose-dihydrate added as cryoprotectant, then freeze-dried at −40 °C and 0.013 mbar for 12 h. Secondary drying was carried out at 25 °C and 0.013 mbar for 3 h. The rehydrated formulation was applied for the Box–Behnken factorial design, micelle characterization and solid-state studies to focus on the effect of MEL and Soluplus^®^. Freeze-dried products were applied for all the investigations for stability studies, nasal product characterization and in vitro studies.

In order to optimize the formulation of MEL-containing polymeric micelles a 3-factor, 3-level Box–Behnken factorial experimental design was set up. The independent variables were, namely the following: the concentration of Soluplus^®^ (mg/mL), the volume of ethanol (mL) and of 1-M NaOH solution (mL) as three critical parameters in the formulation. Based on the factorial design the ethanolic solution before vacuum evaporation contained the concentration of Soluplus^®^ ranged from 6 to 12 mg/mL, the volume of ethanol from 5 to 10 mL and 1-M NaOH solution from 3 to 6 mL (Table 2). Formulation was prepared in triplicate for parallel measurements. The effect on polydispersity index (PdI) and Z-average values was investigated before freeze-drying to investigate the effect of composition without cryoprotectant analyzing the quadratic response surface and to construct a second-order polynomial model using TIBCO Statistica^®^ 13.4 (Statsoft Hungary, Budapest, Hungary) the relationship of the variables on the response could be described with the following second-order equation:

(1) $$Y=\beta_{0}+\beta_{1}x_{1}+\beta_{2}x_{2}+\beta_{3}x_{3}+\beta_{12}x_{1}x_{2}+\beta_{13}x_{1}x_{3}+\beta_{23}x_{2}x_{3}+\beta_{11}x^{2}{}_{1}+\beta_{22}x^{2}{}_{2}+\beta_{33}x^{2}{}_{3},$$

where Y is the response variable; β_0_ is a constant, β_1_, β_2_, β_3_ are linear coefficients, β_12_, β_13_, β_23_ are interaction coefficients between the three factors; and β_11_, β_22_, β_33_ are quadratic coefficients. The analysis of variance (ANOVA) statistical analysis was carried out and the results were evaluated in harmony with their *p*-value when we considered a variable significant if *p* was less than 0.05 at 95% confidence level. Response surface plots for PdI and Z-average in the form of contour plots were plotted according to the regression model by keeping one variable at the center level.

### 2.4. Micelle Characterization

#### 2.4.1. Particle Size Analysis

The average hydrodynamic diameter (Z-average), PdI and zeta potential were measured via dynamic light scattering (DLS) using a Malvern Zetasizer Nano ZS (Malvern Instruments, Worcestershire, UK). The formulations were dissolved in purified water, then measured at 25 °C in folded capillary cells with the refractive index of 1.72. Each measurement was carried out in triplicate with independent formulations. Our criteria were that the particle size (which is closely related to Z-average in stable suspensions) should range from 80 to 120 nm, the PdI less than 0.2 defined by the QTPP with a proper negative zeta potential.

#### 2.4.2. Morphology

The morphology of the freeze-dried (explained in 2.5.6.) MEL-containing polymeric micelles was characterized using transmission electron microscopy (TEM). The TEM records were captured with FEI Tecnai G2 X-Twin HRTEM (FEI, OR, USA) operating at 200 kV accelerating voltage. Suspensions were prepared from the formulations with ethanol then spread to a copper grid coated with a 3-mm-diameter carbon film. For particle size and distribution analysis, a public domain image analyzer software, ImageJ was used (https://imagej.nih.gov/ij/index.html). One hundred particles were measured via the software and the percentage distribution of the particle size was plotted against the particle size. The criterion for particle size distribution was that it is monodisperse when the percentage distribution of one particle size range is greater than 60%.

#### 2.4.3. Thermodynamic Solubility

The thermodynamic solubility of MEL and MEL-containing polymeric micelles was determined in purified water (pH = 7.02; κ = 0.05 µS/cm) at 25 °C, in SNES buffer (pH = 5.6) at 30 °C and PBS buffer (pH = 7.4) at 35 °C to present the pH and temperature conditions of the nasal mucosa and blood vessels. One milliliter of liquids was measured, and products were dissolved until visible oversaturation. After the stirring of the mixtures with a magnetic stirrer for 24 h, they were filtrated through 0.22-µm PES membrane and the content of the dissolved drug was determined with HPLC.

Using the data received from the solubility test the following parameters were calculated [39]:

1. Molar solubilization capacity (χ) or moles of drug that can be solubilized per mol of copolymer forming micelles.
  (2) $$\chi \;=\frac{S_{\mathrm{tot}}-{\;S}_{w}}{C_{\mathrm{copol}}-\;\mathrm{CMC}},$$
2. Micelle–water partition coefficient (P), which is the ratio of the drug concentration in the micelle to the drug concentration in water.
  (3) $$P\;=\frac{S_{\mathrm{tot}}-{\;S}_{w}}{S_{w}}$$
3. Standard free energy of solubilization (∆G_s_^0^), estimated from the molar micelle–water partition coefficient (P_M_).
  (4) $$∆G_{s}^{0}=-\;\mathrm{RT}\cdot \ln\frac{\chi \cdot \left(1-\;\mathrm{CMC}\right)}{S_{w}}=-\;\mathrm{RT}\cdot \ln\left(P_{M}\right),$$

In the equations S_tot_ means the total solubility of MEL in the micellar solution, S_w_ is the solubility of MEL in water, CMC is the critical micelle concentration, C_copol_ is the copolymer concentration in each micellar solution and R is the universal constant of gases.

#### 2.4.4. Encapsulation Efficiency

To determine the encapsulation efficiency for the optimized formulation, we used the indirect method. The MEL-containing polymeric micelles were separated from the aqueous media via centrifugation using a Hermle Z323 K high performance refrigerated centrifuge (Hermle AG, Gosheim, Germany) at 22,500 rpm at 4 °C for 45 min. The clear supernatant was diluted 10-fold with purified water. Quantitative measurements of MEL were performed using HPLC. The encapsulation efficiency (EE%), as the actual MEL content in the optimized formulation, was calculated from this equation:

(5) $$\mathrm{EE}\%\;=\frac{\mathrm{initial}\;\mathrm{MEL}\;\left(\mathrm{mg}\right)-\;\mathrm{measured}\;\mathrm{MEL}\;\left(\mathrm{mg}\right)}{\mathrm{initial}\;\mathrm{MEL}\;\left(\mathrm{mg}\right)}\cdot \;100,$$

#### 2.4.5. Quantitative Analysis of MEL Using HPLC

The determination of MEL concentration was performed with high performance liquid chromatography (HPLC) using an Agilent 1260 (Agilent Technologies, Santa Clara, CA, USA). As stationary phase a Kinetex^®^ C18 column (5 µm, 150 mm × 4.6 mm (Phenomenex, Torrance, CA, USA)) was used. Ten microliters of the samples was injected to determine the concentration of MEL. The temperature was set at 30 °C. The mobile phases used were 0.065-M KH_2_PO_4_ solution adjusted to pH = 2.8 with phosphoric acid (A) and methanol (B). The separation was performed in two steps by gradient elution. The proportion of starting 50% A eluent was reduced to 25% in 14 min and then raised again to 50% in 20 min. The eluent flow rate was set at 1-mL/min and the chromatograms were detected at 355 ± 4 nm using UV–Vis diode array detector. Data were evaluated using ChemStation B.04.03. Software (Agilent Technologies, Santa Clara, CA, USA). The retention time of MEL was at 14.34 min. The linear regression of the calibration line was 0.999. The determined limit of detection (LOD) and quantification (LOQ) in the case of MEL were 16 ppm and 49 ppm.

#### 2.4.6. Surface Free Energy and Polarity Investigation

OCA Contact Angle System (DataPhysics OCA 20, DataPhysics, Inc., GmbH, Filderstadt, Germany) was used for studying the wettability of the polymeric micelles and its components. For the measurements 0.10 g of powder containing the components was compressed under a pressure of 1 t by a Specac^®^ hydraulic press (Specac, Inc., Fort Washington, PA, USA). The liquid mediums used for our contact angle measurements included bidistilled water (interfacial tension of polar component (γ_i_^p^) = 50.2 mN/m, interfacial tension of disperse component (γ_i_^d^) = 22.6 mN/m) and diiodomethane (γ_i_^p^ = 1.8 mN/m, γ_i_^d^ = 49 mN/m). The contact angles of pressings were determined applying the method of Wu. The solid surface free energy is the sum of the polar (γ_i_^p^) and nonpolar (γ_i_^d^) components and was calculated according to the Wu equation [40]:

(6) $$\left(1+\;\cos\Theta \right)\gamma_{l}=\frac{4\left(\gamma_{s}^{d}\gamma_{l}^{d}\right)}{\gamma_{s}^{d}\gamma_{l}^{d}}+\frac{4\left(\gamma_{s}^{p}\gamma_{l}^{p}\right)}{\gamma_{s}^{p}\gamma_{l}^{p}},$$

where Θ is the contact angle, γs is the solid surface free energy and γl is the liquid surface tension. The percentage polarity can be calculated from the γp and γ values:

(7) $$\mathrm{Percentage}\;\mathrm{polarity}\;\left[\%\right]=\frac{\gamma^{p}}{\gamma }\cdot 100,$$

### 2.5. Characterization of MEL–Soluplus^®^ Interaction in Solid State

#### 2.5.1. X-Ray Powder Diffraction (XRPD)

Using X-ray powder diffraction, the changes in the crystalline structure were investigated. The formulations were measured with a Bruker D8 Advance X-ray powder diffractometer (Bruker AXS GmbH, Karlsruhe, Germany) with Cu K λI radiation (λ = 1.5406 Å) and a VANTEC-1 detector. The samples were scanned at 40 kV voltage and 40 mA amperage. The angular range was 3° to 40° 2θ, at a step time of 0.1 s and a step size of 0.007°. All manipulations and evaluations were carried out using EVA software.

#### 2.5.2. Differential Scanning Calorimetry (DSC)

DSC measurements were performed in order to investigate the encapsulation of MEL inside the polymeric micelle. Examinations were carried out using a METTLER-Toledo 821e DSC (Mettler-Toledo GmbH, Gießen, Germany) at the temperature interval of 0–300 °C with the heating rate of 10 °C/min under a constant argon flow of 150 mL/min. Every measurement was normalized to the sample size and was evaluated with STARe Software.

#### 2.5.3. Thermogravimetry (TGA)

TGA measurements were carried out in order to investigate the thermal stability of polymeric micelles. Investigations were performed with a METTLER-Toledo TGA/DSC 1 (Mettler-Toledo GmbH, Gießen, Germany). Then, 5 ± 0.2-mg was measured into aluminum pans, closed and inserted into the furnace. The furnace was heated up from 25 °C to 275 °C with 10 °C/min heating rate and we measured the thermic changes in the sample. The results were evaluated using STARe Software.

#### 2.5.4. Fourier Transform Infrared (FT-IR) Spectroscopy

FT-IR spectroscopic study was performed using an Avatar 330 FT-IR spectrometer (Thermo Nicolet, Waltham, MA, USA) to investigate the interactions between MEL and Soluplus^®^. Pastilles from the samples and 0.15 g potassium bromide were formed using a Specac^®^ hydraulic press (Specac, Inc., USA) by 10 t pressing force. The infrared spectra of the pastilles were collected in the range of 400–4000 1/cm with deuterated triglycine sulfate detector. The spectral resolution was set at 4 1/cm and 128 parallel scans were averaged.

#### 2.5.5. Raman Spectroscopy

For the investigation of polymeric micelles, a Thermo Fisher DXR Dispersive Raman instrument (Thermo Fisher Scientific, Inc., Waltham, MA, USA) equipped with a CCD camera and a diode laser operating at a wavelength of 780 nm was used. Raman measurements were carried out with a laser power of 12 mW at 25-µm slit aperture size with an exposure time of 2 and acquisition time of 6 s, for a total of 32 scans per spectrum in the spectral range 3500–200 1/cm with cosmic ray and fluorescence corrections. The distribution of MEL was investigated by Raman chemical mapping in the formulation. A 90 µm × 90-µm size surface were analyzed with step size of 10 µm with an exposure time of 2 s and acquisition time of 4 s, for a total of 4 scans per spectrum. The Raman spectra were normalized in order to eliminate the intensity deviation between the measured areas.

#### 2.5.6. Physical-Stability via Freeze-Drying

Polymeric micelles are fragile dynamic systems; therefore, it is highly desirable to stabilize the nanoparticles by freeze-drying. In accordance with the ICH Q1A guideline we conducted a stability study in a refrigerator for 12 months at 5 °C ± 3 °C [41]. Each month a portion was dissolved in purified water and then particle size and PdI were measured using the Section 2.4.1 method.

### 2.6. Residual Organic Solvent Determination

Ethanol belongs to Class 3 solvents; thus its residual concentration should be less than 5000 ppm in the daily dose of the final product according to the requirement of the International Council of Harmonization (ICH) Q3C (R5)) guideline for residual solvents [42]. For safety and quality profiling the residual ethanol content of the dissolved polymeric micelles was analyzed by a Shimadzu GC-14B gas chromatograph (Shimadzu Europa GmbH, Duisburg, Germany) equipped with a thermal conductivity (TCD) and a flame ionization detector (FID). The calibration curve was previously determined in the range of 0– 0.35 mM of ethanol. The concentration of ethanol (c_ethanol_) is directly proportional to the area of the peak for ethanol (E_thanol_):

(8) $$c_{\mathrm{ethanol}}=\frac{A_{\mathrm{ethanol}}}{36613992.1},$$

### 2.7. Characterization of Ex Tempore Redispersed Nasal Formulation

The main parameters of ex tempore redispersed lyophilized polymeric micelle formulation were characterized for intranasal applicability. The reconstitution time of powder ampoules containing 3.0 mg of MEL was measured after adding 1.0 mL of purified water (pH = 6.53). After reconstitution, the pH of the colloidal solution was measured with WTW^®^ inoLab^®^ pH 7110 laboratory pH tester (Thermo Fisher Scientific, Budapest, Hungary). Particle size, PDI and zeta potential were determined with dynamic light scattering as described above. The osmolality of the redispersed polymeric micelles was measured by means of an automatic osmometer (Knauer Semi-micro Osmometer, Berlin, Germany) in three parallels. The determination of osmolality is based on the measurement of the freezing point depression of the colloidal solution. Viscosity measurements were performed at 35 °C nasal temperature with a RheoStress 1 HAAKE instrument (Karlsruhe, Germany) conducted with cone–plate geometry (radius 49.9 mm, 1° angle and 0.052 mm gap). The apparent viscosity of the samples was measured over a shear rate sweep of 0.01–100 s^−1^.

### 2.8. In Vitro Characteristics

#### 2.8.1. In Vitro Dissolution Test

The modified paddle method (Hanson SR8 Plus (Teledyne Hanson Research, Chatsworth, CA, USA) was used to examine the rates of dissolution of the polymeric micelle formulations and determine the drug release profile from the samples. One hundred milliliters of SNES was used as a dissolution medium at 35 °C. The paddle was rotated at 100 rpm, and the sampling was performed up to 60 min. Three parallel measurements took place with the optimized formulations. Quantification of aliquots was performed by HPLC after filtration with the method described previously.

From the data acquired from the dissolution test, the following statistical analysis of MEL dissolution profile was evaluated [32]. The percentage dissolution efficiency (%DE) for MEL and each MEL-containing polymeric micelle formulation was calculated as the percentage ratio of the area under the dissolution curve up to time t to that of the area of the rectangle described by 100% dissolution at the same time as follows:

(9) $$\%\mathrm{DE}=\frac{\int_{0}^{t}y\;\cdot \;\mathrm{dt}}{y_{100}\;\cdot \;t}\;\cdot 100\%,$$

The trapezoidal method was used to calculate the area under the curve (AUC_0—t_) which is the sum of all the trapezia defined by:

(10) $$\mathrm{AUC}=\overset{i=n}{\underset{i=1}{\sum }}\frac{\left(t_{1}-t_{i-1}\right)\left(y_{i-1}+y_{i}\right)}{2},$$

where t*_i_* is the time point and *y_i_* is the percentage of product dissolved at time t*_i_*.

The relative dissolution (RD) time at 15 min (RD_15_) in the case of the formulations compared to crystalline MEL was calculated using the formula:

(11) $${\mathrm{RD}}_{15\;min}=\frac{{\%\mathrm{DE}}_{15\;\min}\mathrm{Pol}\_\mathrm{mic}}{{\%\mathrm{DE}}_{15\;\min}\mathrm{MEL}},$$

The mean dissolution time (MDT) was calculated using this expression:

(12) $$\mathrm{MDT}=\frac{\sum_{i-1}^{n}t_{\mathrm{mid}}∆M}{\sum_{i-1}^{n}∆M},$$

where *i* is the dissolution sample number, *n* is the number of dissolution times, t_mid_ is the time at the midpoint between times t*_i_* and t*_i_*_−1_ and Δ*M* is the amount of MEL dissolved (mg) between times t*_i_* and t*_i_*_−1_.

#### 2.8.2. In Vitro Nasal Diffusion

In vitro nasal diffusion study of MEL from the nasal cavity was performed in a modified Side-Bi-Side^®^ horizontal diffusion cell. A cellulose membrane impregnated with isopropyl myristate having a surface of 0.785 cm^2^, the cell volume was 9.0 mL and the diffusion was investigated at 35 °C. The donor phase consisted of pH 5.6 SNES solution simulating the nasal fluid and the acceptor phase was a pH 7.4 phosphate buffer simulating the plasma in the nasal vessels. Absorbance was inline measured in each 50 ms for 60 min on an AvaSpec 204 BB spectrophotometer (Avantes, Apeldoorn, The Netherlands) at 364 nm in the acceptor phase. Three parallel measurements took place with the optimized formulations (Pol_mic1–3). The flux (J) of the drug was calculated from the quantity of MEL which permeated through the membrane, divided by the surface of the membrane insert and the duration (µg/cm^2^/h) [43]. The permeability coefficient (K_p_) was determined from J and the drug concentration in the donor phase (C_d_ (µg/cm^3^):

(13) $$K_{p}\left[\frac{\mathrm{cm}}{h}\right]=\frac{J}{C_{d}}$$

Relative permeability (RP) at 15 min (RP_15_) in the case of the formulations compared to crystalline MEL was calculated using this formula:

(14) $${\mathrm{RP}}_{15\;\min}=\frac{{\mathrm{Diffused}\;\mathrm{MEL}}_{15\;\min}\mathrm{Pol}\_\mathrm{mic}}{{\mathrm{Diffused}\;\mathrm{MEL}}_{15\;\min}\mathrm{MEL}}$$

where the diffused MEL values were calculated from the slopes between 10 and 20 min.

#### 2.8.3. In Vitro and In Vivo Correlation (IVIVC)

According to the FDA guidance [34,35,36], an IVIVC is defined as a predictive mathematical model describing the relationship between an in vitro property of a dosage form and an in vivo response. Thus, IVIVC can be used to predict the in vivo pharmacokinetics of the formulation from its in vitro dissolution and diffusion data. In contract, it can also help to design the optimal in vitro dissolution and diffusion profiles of the formulation for the desired in vivo pharmacokinetics. Therefore, once the predictability of IVIVC has been established, the in vitro dissolution and diffusion may provide a surrogate for in vivo experiments [36]. Among the different levels of IVIVC, a level-C IVIVC allows for predicting the relationship between in vivo pharmacokinetic parameters (e.g., API brain distribution– AUC) and in vitro data (dissolution rate, diffusion rate) at a single point, but it does not substitute bioequivalence study. As IVIVC is generally described by a linear relationship between parameters derived from the in vitro and in vivo experiments, the Pearson correlation was applied to investigate that relationship. Previous in vitro and in vivo data of different MEL containing nasal formulations with equal drug content was used for IVIVC to predict the in vivo brain distribution of intranasal administration of MEL-containing polymeric micelles. Data were expressed as means ± SD, and groups were compared by Student’s t-test using TIBCO Statistica^®^ 13.4 (Statsoft Hungary, Budapest, Hungary). Differences were considered statistically significant when *p* < 0.05.

## 3. Results

### 3.1. Initial Risk Assessment and Knowledge Space Development

QTTP was defined as Soluplus^®^-based polymeric micelles containing MEL, as a nonsteroidal anti-inflammatory drug, with proper particle size and distribution and optimal physicochemical profile. The formulations should ensure proper dissolution and permeability parameters, in order to reach the central nervous system via intranasal administration bypassing the first-pass effect, which can be applied by an adult population suffering from neurodegenerative diseases. Due to the fact, that polymeric micelles are one of the nanodrug delivery systems, a special regulatory aspect is needed to exclude potential nanotoxicity. This needs further information on safety, efficacy and the quality of the product in the submissions during the authorization process. By targeting the brain via this administration route, we must also consider avoiding systemic side effects. Risks can originate from the final product itself, e.g., physical-stability, dissolution and permeability issues, solubility and polarity as well as from the therapeutic use (irritation, damage of the nasal mucosa, effects on nasal function, e.g., smell). The patient could belong to a risk group during administration (lack of adherence, increased effect with side effects due to fast dissolution).

**Figure 1:**
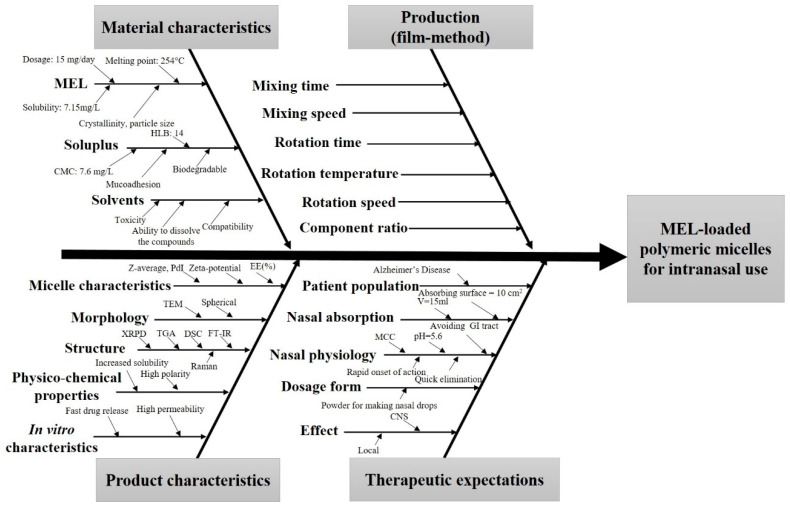
Ishikawa—or cause and effect diagram—of the influencing parameters of meloxicam (MEL)-containing polymeric micelles for intranasal use. Abbreviations not mentioned before: HLB—hydrophile–lipophile balance; GI—gastrointestinal; MCC—mucociliary clearance.

During the knowledge space development, which is the collection of the influencing factors, the potential factors which can influence the final product were grouped into four sections based on the classic 4-M Ishikawa (or cause and effect) diagram: material characteristics, production method, product characteristics and therapeutic expectations (Figure 1).

**Figure 2:**
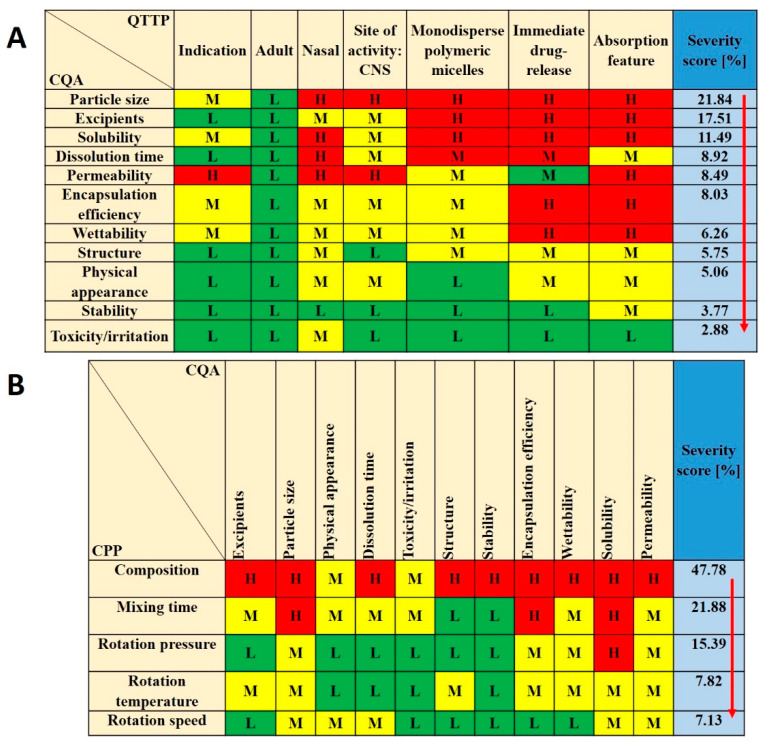
Risk assessment of the MEL-loaded polymeric micelles. (**A**,**B**) Interdependence ratings between (**A**) CQAs and QTTPs and CQAs and (**B**) CPPs with the calculated severity scores of CQAs and CPPs in decreasing order of risks. Abbreviations: QTPP—quality target product profile; CQA—critical quality attributes; CPP—critical process parameters; L—low; M—medium; H—high.

For CPPs the following were selected: composition, mixing time, rotation pressure, temperature and speed. These parameters were followed carefully during the formulation and became one of the bases for further factorial experimental design. CQAs were the following: particle size, excipients, encapsulation efficiency (EE%), dissolution time, solubility, permeability, wettability, structure, stability, physical appearance and toxicity/irritation, as these factors have critical effects on the QTPP. Among the QTPP elements, CQAs and CPPs an initial RA was performed (Figure 2).

Tables of Figure 2 present the estimated relationships of the selected critical factors. Three levels were assigned for each interaction. The ones that have minimal effect on each other are marked green, the interactions with medium effect are marked yellow and the ones with the highest impact are marked red. Using the Lean QbD^®^ Software, the severity score was determined, and the factors were ranked from highest to lowest based on their impact. It was found that, theoretically, particle size, excipients, dissolution time, EE%, solubility and permeability had the highest impacts among CQAs on the quality and efficacy of MEL-loaded polymeric micelles. The analogous diagram shows that among CPPs, the composition of the formulation was expected to have the highest influence. The other CPPs can be easily adjusted and may have a minimal effect on the quality of the final product. The results of the RA gave the basis of the factorial experimental design which was built up based on variations of the composition. As for CQAs, particle size (expressed as Z-average) and particle size distribution (expressed as PdI) can be primarily optimized with the appropriate composition. They have a direct impact on EE%, solubility, dissolution and permeability, which were later investigated as well.

### 3.2. Production and Optimization of MEL–Soluplus^®^ Polymeric Micelles via Box–Behnken Factorial Design

Using the experimental design, the ratio of Soluplus^®^ and ethanol with the excipient 1-M NaOH solution was investigated. After rotary evaporation and vacuum drying, the films were rehydrated with 6 mL of purified water and Z-average and PdI were measured in triplicate with independent formulations. The results from the 15 formulations were analyzed by TIBCO Statistica 13.4 software and are shown in Table 2.

Polynomial equations were generated to describe the individual main effects and interaction effects of the independent variables on the dependent factors individually. According to the ANOVA and regression analysis of the data, the relationship of the variables on PdI (Y_1_) could be described with the following equation:

Y_1_ = 0.319 + 0.084x_1_ − 0.025x_2_ + 0.003x_3_ + 0.118x_1_x_2_ − 0.027x_1_x_3_ + 0.077x_2_x_3_ + 0.084x^2^_1_ − 0.025x^2^_2_ − 0.030x^2^_3_. (15)

The regression coefficient of the surface plot was 0.998 (R^2^), the adjusted was 0.989 (R^2^) which indicates good correlation. The concentration of Soluplus^®^ (x_1_) and the volume of ethanol (x_2_) affected (*p* < 0.05) the PdI significantly. The positive coefficients before the independent variables (x_1_, x_3_, x_1_x_2_, x_2_x_3_, x^2^_1_) are unfavorable, because it means that by increasing the value of the variables, we increase the PdI while the negative coefficients (x_2_, x_1_x_3_, x^2^_2_, x^2^_3_) decrease it. The proper concentration of the polymeric micelle forming agent in the right volume of ethanol solvent resulted in favorable formulation. Our criteria were to reach PdI less than 0.2 with a Z-average ranging from 80 to 120 nm. Increasing the concentration was unfavorable for PdI because the higher the concentration, the more excess polymer dissolves, which results in unloaded, blank polymeric micelles. These have lower Z-average resulting in high PdI compared to the MEL-loaded ones with monodisperse distribution.

The effect of the independent factors on Z-average (Y_2_) can be described using the following equation:

Y_2_ = 150.94 + 40.52x_1_ − 7.03x_2_ − 5.09x_3_ + 45.43x_1_x_2_ + 4.78x_1_x_3_ + 29.38x_2_x_3_ − 28.63x^2^_1_− 13.53x^2^_2_ + 5.87x^2^_3_. (16)

**Figure 3:**
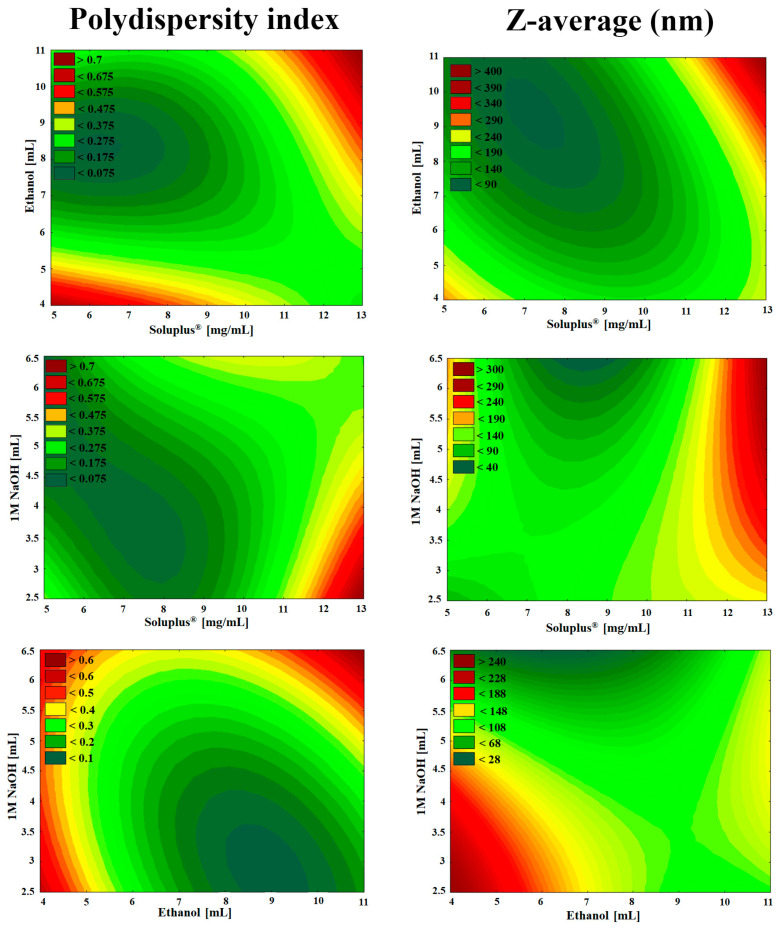
Two-dimensional surface plots of the effect of independent variables in the Box–Behnken factorial design.

The regression coefficient of the surface plot was 0.999 (R^2^), the adjusted was 0.993 (R^2^), which also indicates good correlation. The concentration of Soluplus^®^ (x_1_) and the interaction between the concentration of Soluplus^®^ and the volume of ethanol (x_1_x_2_) were significant (*p* < 0.05). The positive coefficients before the independent factors (x_1_, x_1_x_2_, x_1_x_3_, x_2_x_3_, x^2^_3_) result in increased particle size which also clashes with our criteria that the particle size (expressed in Z-average, therefore in hydrodynamic diameter) should be in the range of 80 to 120 nm. The negative coefficients before the factors (x_2_, x_3_, x^2^_1_, x^2^_2_) decrease the particle size. The contour plots in Figure 3 also indicate the proper amount of independent factors.

As seen in the Box–Behnken factorial design, the volume of 1 M NaOH solution was not linearly significant either in the cases of PdI or Z-average. The high melting point of MEL (254 °C), thermal stability and the glass transition temperature of Soluplus^®^ (70 °C) allowed a safe and controllable formulation. As a result, solid MEL-containing polymer micelles were obtained which dissolve rapidly in small volumes of purified water. The solution visibly showed a bluish opalescence (Tyndall effect) which is a characteristic of the colloidal, especially the nanoscale systems. Based on the design space (Figure 3 green area) the optimized composition of formulation contained 2.5-mg/mL MEL, 9-mg/mL Soluplus^®^, 10 mL Ethanol and 3 mL NaOH with suitable Z-average (111.6 ± 3.0), monodisperse size distribution (PdI of 0.114 ± 0.06) and the zeta potential value was −25.2 ± 0.4 mV. This surface charge prevents the aggregation of the nanoparticles, i.e., they are dispersed stably and meet the physicochemical criteria. This optimized formulation was applied for further characterization. In case of formulations having large PdI there was a mixture of subpopulation of different sizes. These compositions showed heterogeneous size distribution, a mixture of subpopulation of different sizes with a peak at 80 nm corresponding to unloaded micelles and a peak with the maximum higher than 500 nm referred to aggregation of micelles may occur.

### 3.3. Micelle Characterization

#### 3.3.1. Morphologic Characterization

**Figure 4:**
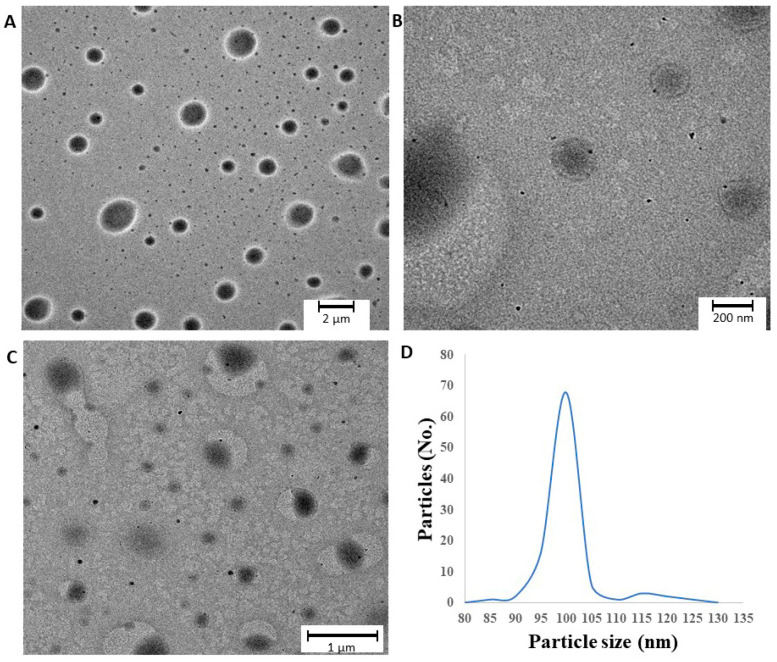
(**A**–**C**) TEM images of optimized freeze-dried MEL-loaded polymeric micelles and (**D**) particle size distribution.

TEM images and the particle size distribution of rehydrated polymeric micelles are shown in Figure 4. The polymeric micelles are spherical and have a smooth surface. They feature a quasi-monodispersed diameter distribution with particle size ranging from 85 to 135 nm which corresponds to our DLS measurements explained above. The previously set criterion was fulfilled, with particle distribution of 68% in the range of 100 to 105 nm. In the images suspended d-trehalose dihydrate bigger in diameter can be detected as well, which was excluded from the particle size analysis.

#### 3.3.2. Encapsulation Efficiency and Solubility Studies

Table 3 summarizes the parameters which were used to quantify the efficiency of solubilization. The solubility test showed that the solubility values of the MEL-containing polymeric micelles are much higher compared to the crystalline MEL in all three media. The solubility increases parallel with the pH, as MEL is a weak acid (pKa = 4.08), therefore its solubility is higher in the weakly alkaline environment. The polymeric micelles follow the same tendency. The micelle–water partition coefficient recorded was highly above 1, ranging from 782.20 to 825.45, and the P_M_ value ranged from 77.68 to 81.98, which means that there are more MEL molecules localized in the core of the polymeric micelles than free (solubilized) ones. These results confirmed efficient incorporation supported by negative free energy of solubilization, which resulted in spontaneous association and was thermodynamically favored by the hydrophobicity of Soluplus^®^ polymeric micelle cores. The results can be explained by the monodispersed, proper particle size decrease because of Soluplus^®^ and the high encapsulation efficiency. The amorphous state and the lack of lattice energy also support these results which could predict the faster dissolution rate and nasal diffusion of MEL-loaded polymeric micelles.

#### 3.3.3. Surface Free Energy and Polarity Investigation

The wettability study showed that the formulations had a more hydrophilic character as compared with crystalline MEL (Table 4). The contact angle of MEL (75.3°) was relatively high, showing its poor hydrophilicity, while the contact angle of formulations was lower (11.3°) predicting higher hydrophilic character. Soluplus^®^ by itself has a good polarity as well. By encapsulating MEL in the polymeric micelle, the surface characteristics and polarity increased. These results corroborate the thermodynamic solubility mentioned earlier because higher polarity presumes higher solubility in water.

### 3.4. Characterization of MEL–Soluplus^®^ Interaction in Solid State

#### 3.4.1. X-Ray Powder Diffraction (XRPD) Study

**Figure 5:**
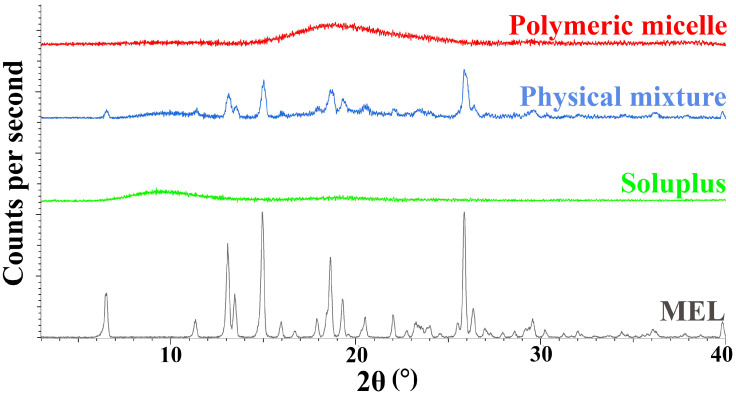
X-ray powder diffractograms of the components and the formulation.

During our experiment, the diffractograms of crystalline MEL, Soluplus^®^, their physical mixture and the polymeric micelles were recorded. The diffractograms in Figure 5, show that the characteristic peaks of crystalline MEL could not be detected in the polymeric micelles compared to the physical mixture components. Based on the results, we can conclude that a new system was formed between MEL and Soluplus^®^ with the encapsulation process, only the amorphous structure of the micelle forming excipient can be observed in the diffractogram of polymeric micelles. MEL itself has a poor glass forming property (T_g_/T_m_ = 0.63), which proves that MEL did not go through amorphization, it was molecularly dispersed into the core of polymeric micelles and the amorphous sign of Soluplus^®^ as micelle shell forming agent suppressed its characteristic peaks [44].

#### 3.4.2. Differential Scanning Calorimetry (DSC) Measurement

**Figure 6:**
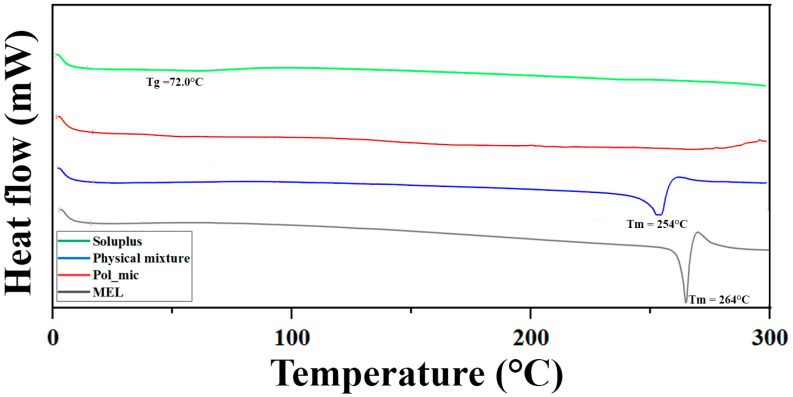
Differential scanning calorimetry (DSC) curves of the components and the formulation.

The DSC curves (Figure 6) support the results of the XRPD study, the endothermic peak corresponding to the melting point of MEL disappears in the formulation, suggesting the encapsulation inside the micelle, unlike in the physical mixture. A slight reduction in the melting point from 264 °C to 254 °C in the physical mixture can be observed, which could occur due to the formation of a solid dispersion.

#### 3.4.3. Thermogravimetric Analysis (TGA)

TGA measurements were carried out to investigate the thermal stability of the formulation. The degradation was examined in the temperature interval between 25 °C to 275 °C. The weight loss of each component was measured, which can be seen in Table 5, showing that the polymeric micelles are stable against temperature. For the polymeric micelles two data were collected, one at 103 °C where it lost almost 1% of its weight and one at 237 °C losing 5% of it. As these are already extreme conditions, both during the storage of the formulation and in the production of polymeric micelles, it is safe to say that the products are stable against temperature increase. The loss of weight corresponds with the reduction of hydroxyl groups of PEG 6000 portion in Soluplus^®^ by condensation.

#### 3.4.4. Fourier-Transform Infrared (FT-IR) Spectroscopy

**Figure 7:**
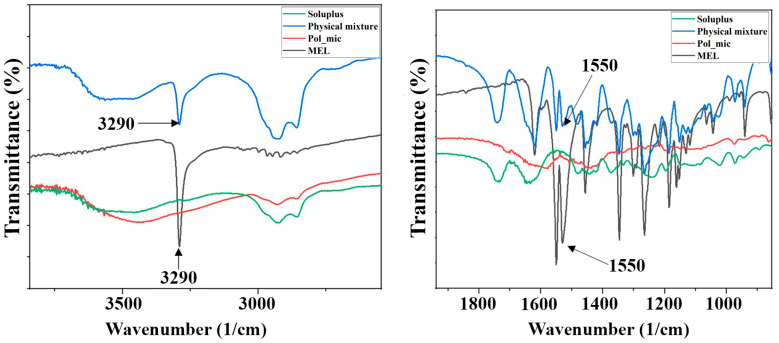
FT-IR spectra of the enlarged regions indicating the secondary interactions.

Hydrogen bonding among polymeric micelle forming components can enhance the intra-micelle interaction and micelle stability. Hydrogen bonding interactions are selective and noncovalent interactions, which only form between hydrogen bonding donors and acceptors. The participation of hydrogen bonding interactions directs the self-assembly of copolymers and stabilizes the micellar cores, which exhibit great potential as nanoDDSs. Vibrational spectroscopy is a suitable tool for the determination of H-bonding. The FT-IR spectra of polymeric micelles and components can be seen in Figure 7.

The spectral peak at 3290 cm^−1^ for the hydroxyl group attached to an aromatic ring of MEL, as well as the vibration of the carbonyl group at 1550 cm^−1^ are not visible in the spectrum of polymeric micelle. The disappearance of the characteristic peaks indicates the formation of a new composition that is, they cannot be described on either the polymer or the MEL. The investigation of the new entity was further characterized using Raman spectroscopy.

#### 3.4.5. Raman Spectroscopy and Chemical Mapping

**Figure 8:**
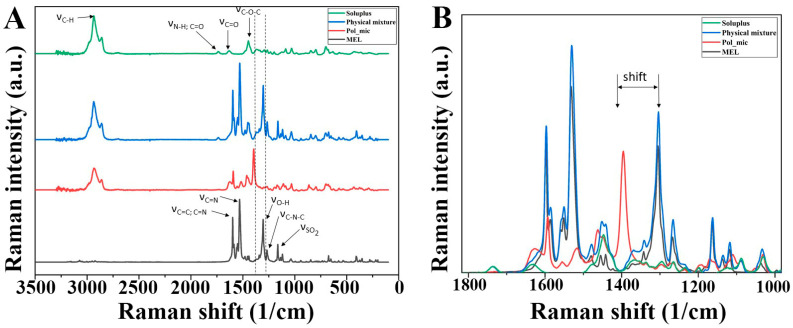
(**A**) Raman spectra of the materials and the (**B**) enlarged region of structural change.

A Raman spectrum of MEL, Soluplus^®^, their physical mixture and MEL-loaded polymeric micelles is shown in Figure 8A. MEL shows bands of aromatic ring vibration of benzothiazine at 1598 cm^−1^, which derives from the stretching vibration of C=C or C=N and the bending vibration of CH_2_. The sharp peak at 1532 cm^−1^ indicates the stretching vibration of C=N in thiazole ring. Other characteristic absorptions appeared at 1305 cm^−1^ (ν_OH_), 1267 cm^−1^ (ν_C–N–C_) and 1164 cm^−1^ (ν_C–S_). The Raman spectrum of the polymeric micelle showed that the band located at 1305 cm^−1^ for starting meloxicam shifted to higher energy at 1394 cm^−1^ as a result of the deprotonation of the molecule. The appearance of a new peak due to the deprotonation of phenolic OH changed the vibrational motion of C=C and C–N bonds [45]. The magnitude of the shift in the spectrum was 89 cm^−1^, which reflects the OH group attached to the six-membered heteroatom ring attends in the H-bond forming with Soluplus^®^ (Figure 8B).

**Figure 9:**
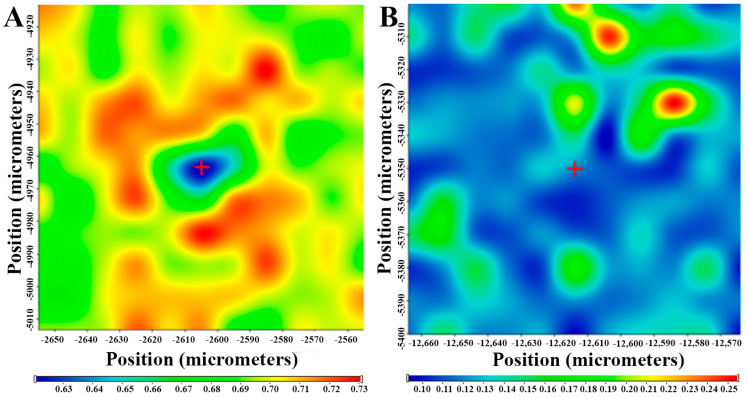
Intensity changes in Raman chemical maps indicating the (**A**) shifting at 1305 nm of physical mixture and (**B**) polymeric micelle; color intensity: blue—extinction of peak at 1305 nm, red—existence of peaks at 1305 nm.

In order to confirm the distribution of H-bonding in the polymeric micellee Raman mapping of the physical mixture and the polymeric micelle was carried out. The characteristic bands of phenolic OH shifting (1394→1305) as highlighted regions were used to visualize with chemical mapping (Figure 9). The chemical maps showed the Raman shift of phenolic OH can be observed on the whole surface of polymeric micelles in contrast to the physical mixture. This phenomenon confirms the existing H-bonding in the polymeric micelles.

#### 3.4.6. Physical-Stability of Freeze-Dried Formulations

Freeze-dried samples were analyzed continuously at specific times during the stability test. The particle size and PdI of aliquots dissolved in purified water were determined as seen in Table 6. To calculate the absolute change, 0th month data were extracted from 12th month, since it has the largest difference from the starting point. The particle size increased by 3.31 nm, while PdI by 0.016 after 12 months of storage. The samples met the requirements previously set, i.e., having a particle size ranging from 80 to 120 nm and a PdI of less than 0.2. The 5 *w/w*% d-trehalose dihydrate was a suitable cryoprotectant for solid storage of freeze-dried MEL-containing polymeric micelles.

### 3.5. Residual Organic Solvent Determination with Gas Chromatography

All new products that are planned to be placed on market must comply with very strict rules on impurities and residual formulation excipients based on The International Council of Harmonization (ICH) Q3C (R5)) guideline for residual solvents [46]. With the film-method we could achieve a formulation having a low concentration of ethanol. As the concentration of the polymer is at the CMC value, polymer chains associate to form micelles in a way that hydrophobic parts avoid contact with the aqueous media, therefore encapsulating some of the non-aqueous media, in our case: ethanol. At CMC we could detect the highest concentration in the micellar core with the largest particle size, but as the solvents evaporate, and the polymer concentration increases above CMC, the equilibrium favors micelle formation. The micelles will take their lowest energy state configuration and the remaining solvent will gradually release from the core. The decrease of particle size will follow this [47]. After evaporation, secondary drying in the vacuum chamber also contributed to the loss of ethanol without damaging or changing the micellar structure in solid form allowing to dissolve the formulations in water. The results can be seen in Table 7.

### 3.6. Characterization of Ex Tempore Redispersed Nasal Formulations

The main parameters influencing nasal absorption were measured after the reconstitution of the lyophilized formulation (Table 8).

The reconstitution time of lyophilized polymeric micelles was 2 s, which meets the requirements of European Pharmacopoeia (Ph. Eur. 10), according to which a lyophilized product reconstituted to a solution using an appropriate diluent prior to patient administration should be less than 3 min. The physiological pH in the nose is 6.4 on average and this allows normal ciliary function. In this slightly acidic environment, lysozyme, the natural antimicrobial agent in the nose, is effective in the prevention of growth of pathogenic bacteria in the nasal passage. A major deviation from this causes irritation of the nasal mucosa. To avoid that, formulation pH should be kept between 4.5 and 6.5 so our formulation satisfies that criterion [48]. The Z-average, PdI and zeta potential values meet the requirements of intranasally administered nanoparticles. The Z-average (100.47 ± 0.87 nm) of polymeric micelles is low enough for fast absorption. The PdI (0.149 ± 0.01) shows monodisperse size distribution, which ensures the homogeneity of polymeric micelles in the aqueous media. The zeta potential (−26.7 ± 0.6 mV) is high enough to avoid aggregation in liquid form through the repulsion among the nanoparticles. The osmolality of the polymeric micelle formulation was hypotonic (240 mOsmol/L). Nasal preparations are normally isotonic (about 290 mOsmol/L), which is also best tolerated, but sometimes a deviation from isotonicity may be an advantage. Hypertonic solutions shrink epithelial cells and inhibit ciliary activity. On the other side, hypotonic solutions can increase drug absorption, as we also expect from our formulation. The resulting viscosity of the preparation will directly affect the droplet size of the spray depending on rheological properties and spray characteristics of the spray plume. The viscosity of the formulation was 32.5 ± 0.28 mPas which is suitable to form intranasal liquid formulation, likely through mild mucoadhesive property [49]. On the other hand, more viscous formulations provide less efficient systemic nasal drug delivery due to slower diffusion [50].

### 3.7. In Vitro Characteristics

#### 3.7.1. In Vitro Dissolution Study

**Figure 10:**
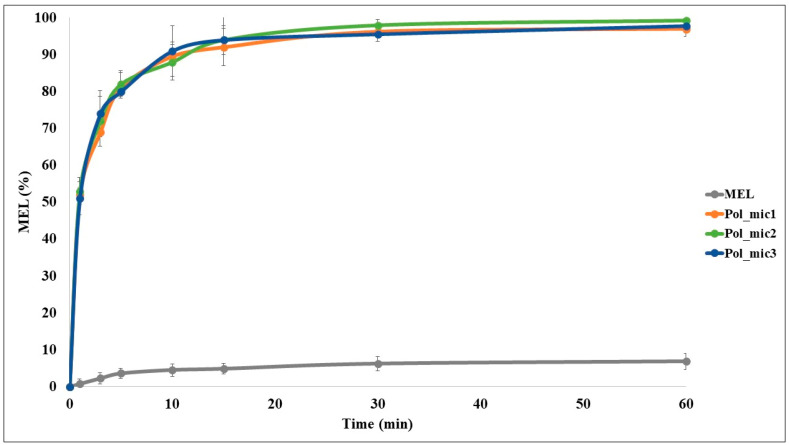
In vitro release study of crystalline MEL and the optimized formulation in triplicate; data are means ± SD (*n* = 3 independent formulations)

The in vitro dissolution test was used to ascertain that the improved rate of dissolution of MEL is achieved as shown in Figure 10. The optimized formulation was examined in 3 parallel measurements. The rate of dissolution of crystalline MEL with particles in the micrometer size range was very slow, only 4.83% of the drug was dissolved in 15 min. Formulation of the MEL-containing polymeric micelles improved greatly the dissolution rate to 93.6% in average in 15 min. This improvement of dissolution can lead to a rapid onset of action. For nasal formulations it is a criterion that the drug must have an almost complete dissolution profile in 15 to 20 min, because of the quick elimination caused by mucociliary clearance (MCC). The increased surface area due to the formulation of nanosized MEL-contained polymeric micelles, the surface-active Soluplus^®^ and the improved wettability and higher polarity may have contributed to the increase. As mentioned before in the introduction the polymeric micelles can release the drug in two ways: either diffusion or breaking into monomers. With the promising results it can be said that these mechanisms can be accomplished on the pH of the nasal cavity ending up in rapid release. As the TEM images showed above, the formulations have a spherical morphology, which is known that a sphere has the highest surface area of all equal volume spatial shapes which factor may also contributes to the increase.

It can be seen in Table 9 that the dissolution efficiency at 30 min was enhanced (from 4.52% to 87.01%) due to the formulation and the same increase could be observed in the case of RD values (from 1.00 to 19.23). The MDT values decreased from 11.50 to 3.59, which means that the dissolution became faster due to the earlier explained reasons.

#### 3.7.2. In Vitro Nasal Diffusion Study

**Figure 11:**
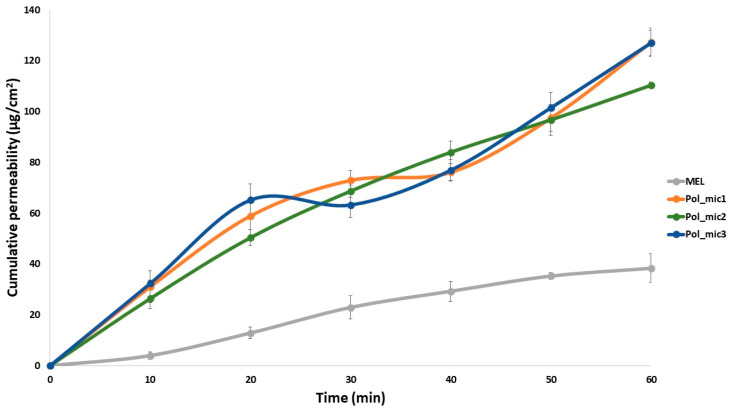
In vitro permeability study of MEL-loaded polymeric micelles in triplicate compared to crystalline MEL; data are means ± SD (*n* = 3 independent formulations).

The cumulative amount of MEL that diffused through the membrane from MEL-loaded polymeric micelles was measured against time on the modified Side-Bi-Side^®^ horizontal diffusion cell (Figure 11). The measurements took place in real time where the absorbance was detected every 50 ms. Three parallel measurements took place from the same formulation. The diffusion from the formulation containing MEL nanoparticles was highly increased compared to crystalline MEL, due to the rapid dissolution of MEL. The diffused MEL ratio was ranging from 72.18% to 83.29% which is higher than crystalline MEL with a value of 25.08%. The results can be explained by the particle size reduction and high solubility and polarity values determined before. The flux (J), which shows the amount of MEL that permeates through the membrane within 1 h increased in case of the polymeric micelles. The permeability coefficient (Kp) showed the same tendency in all three parallel measurements as well (Table 10). The RP_15_ value showed an approximately fivefold increase in the extent of degree of diffusion. These results are a must in the case of intranasal administration, and they are typical of fast diffusing systems.

#### 3.7.3. In Vitro and In Vivo Correlation (IVIVC) of Polymeric Micelles

**Figure 12:**
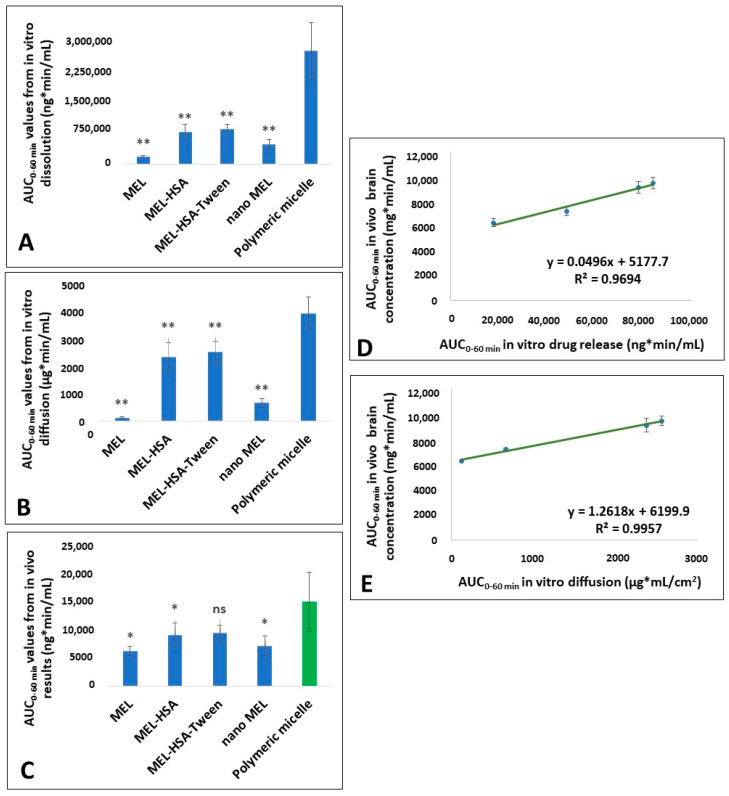
Figures of the IVIVC evaluation. (**A**) Calculated AUC_0–60 min_ values from the in vitro dissolution test; (**B**) Calculated AUC_0–60 min_ values from the in vitro diffusion test; (**C**) AUC_0–60 min_ values from the in vivo results with the predicted polymeric micelle value (marked as green); (**D**) Pearson correlation based on dissolution; (**E**) Pearson correlation based on diffusion. Statistics: * = *p* < 0.05; ** = *p* < 0.01; ns = not significant.

To estimate the statistical in vivo brain distribution of MEL-containing polymeric micelles IVIVC was performed applying in vitro and in vivo data of previous experiments, namely a MEL suspension, MEL-containing human serum albumin nanoparticles with Tween-20 (MEL–HAS–Tween) and without surfactant (MEL-HAS) [18] as well as a MEL-containing nanosuspension [27]. These formulations contained MEL in the same concentration. For investigating the relationship between in vitro and in vivo data, Pearson correlation was used. The Pearson correlation coefficients showed good fit both in the case of in vitro dissolution (0.9694) and diffusion (0.9957). Based on the regression curves, the possible in vivo brain distribution of MEL containing polymeric micelles was estimated to be approximately 15,017 ± 5327 ng∙min/mL. The comparative studies according to AUC_0–60 min_ values of in vitro dissolution, in vitro diffusion and in vivo brain distribution have shown that a significant difference is expected between the measured in vitro and the estimated in vivo results of polymeric micelle formulation and the different MEL-containing intranasal formulations applied for comparison. The in vitro dissolution and diffusion of MEL from polymeric micelles was significantly higher than from the other drug delivery systems, but its expected AUC_0–60 min_ value of in vivo brain distribution, in comparison to MEL-HSA-Tween, showed no significant difference (Figure 12).

## 4. Discussion

The aim of this study was to formulate MEL-loaded Soluplus^®^ polymeric micelles for intranasal application to target the brain using a variation of the film-method or otherwise known as the rotary-evaporation method. As neuroinflammation plays a pivotal role in the progression of neurodegenerative diseases, the nose-to-brain administration of NSAIDs in a smart nanoDDS can be advantageous. Previous studies described improved bioavailability of nose-to-brain administered polymeric micelles containing CNS targeted drugs such as sumatriptan [51] or clonazepam [52]. An improved anti-neuroinflammatory effect was experienced in case of monoterpenoids like geraniol charged polymeric micelles [53], but intranasal NSAID-containing polymeric micelle formulations were not investigated yet, therefore it is considered a novel way of application.

The formulation was developed using a QbD driven factorial experimental design. Polymeric micelles were prepared using the film method by the rotary evaporation of solution containing MEL and Soluplus^®^ dissolved in ethanol and sodium hydroxide solution. The optimal MEL-Soluplus^®^ ratio was approximately 1:4. The polymeric micelles were formed after rehydration of the matrix film by addition of purified water. The main advantage of this method is that the organic solvent can be practically completely removed and may also result in a significant increase in drug loading [54]. The technology used is scalable, environmentally friendly and also meets the requirements of ICH in terms of residual solvent [46]. In the formulation study the average particle size, polydispersity index (PdI) and zeta potential had the highest impacts among critical quality attributes (CQAs) on the quality and efficacy of MEL-loaded polymeric micelles.

The critical product parameters of reconstituted polymeric micelles, such as Z-average (100.47 ± 0.87 nm) of polymeric micelles was low enough for fast absorption [55] and PdI (0.149 ± 0.01), shows monodisperse size distribution, ensuring to overcome recognition and removal by the reticuloendothelial system (RES) [56]; moreover, the zeta potential (−26.7 ± 0.6 mV) also met the requirements of nano DDSs ensuring monodispersity through the high charge–charge repulsion among the particles [57]. The Soluplus^®^ as micelle-forming agent ensured a good wettability attribute and polarity (47.0%) leading to high solubility (5419.7 ± 1.284 mg/L) and high encapsulation efficiency (89.4%) at pH 5.6.

The developed MEL-containing liquid micellar delivery system was converted to a solid phase product by freeze-drying using D-trehalose dihydrate in five mass percent as a cryoprotectant agent to stabilize the system. After 12 months of storage the particle size remained decent: 103.78 ± 0.36 nm with a PdI of 0.165 ± 0.04, the particle size increased only by 3.31 nm, while PdI by 0.016 based on the DLS and TEM results. The product met the requirements previously set, i.e., having a particle size ranging from 80 to 120 nm and a PdI of less than 0.2. The five mass percent D-trehalose dihydrate was a suitable cryoprotectant for solid storage of freeze-dried MEL-containing polymeric micelles. DSC and XRPD results confirmed the amorphous nature of polymeric micelles which also contributes to the solubilization of MEL. TGA measurements revealed the relatively high thermal stability of the nanoDDS, the formulation remained stable as the temperature increased up to 103 °C. FT-IR and Raman investigations showed interactions were formed between Soluplus^®^ and MEL stabilizing the drug delivery system.

The reconstitution time of ex tempore reconstituted polymeric micelles was only two seconds, which met the requirements of Ph. Eur. 10 (<3 min). Other nasal formulation characterization studies were also carried out recommended by the EMA [58]. The pH of liquid formulation was 6.49, which allows normal ciliary function [59]. The Z-average, PdI and zeta potential values meet the requirements of intranasally administered nanoparticles. The osmolality of the polymeric micelle formulation was hypotonic (240 mOsmol/L), which can increase drug absorption [60], as we also expect from our formulation. The viscosity of the formulation was 32.5 ± 0.28 mPas which is suitable to form intranasal liquid formulation possibly through mild mucoadhesive property [49].

The in vitro dissolution and permeability experiments also fulfills the criteria of intranasal administration having high dissolution (%DE_15 min_ = 79.04 ± 0.454) and permeability (121.56 ± 9.73 µg/cm^2^/h) values at 15 min supported by the statistical analysis. Five-fold higher nasal permeability and more than 20-fold faster drug dissolution was reached by polymeric micelles in comparison to starting MEL.

The experimental design and the in vitro dissolution and permeability results were focused on the IVIVC to estimate the potential in vivo brain distribution of MEL. Based on the results, a good brain distribution for intranasal application of the developed formulation is expected to be approximately 15,017 ± 5327 ng∙min/mL. In the early stage development IVIVC is also justified as regulatory guideline in order to minimize the number of further animal studies [61]. The IVIVC results of polymeric micelles suggest the great potential for in vivo brain distribution of MEL. The nose-to-brain delivery of NSAIDs such as MEL using nanoDDS as polymeric micelles may provide a new opportunity to treat neuroinflammation more effectively.

## 5. Conclusions

A QbD driven initial product design study of MEL-loaded Soluplus^®^ polymeric micelles provides a new possibility of intranasal NSAID administration against neuroinflammation, which was not described previously. In this approach a “value-added” solid phase viscosity enhancer- and preservative-free nasal product can be successful developed, which can be easily converted “ex tempore” to a liquid product and is ready-to-use as a nasal drop or spray and may substitute current therapeutics.

## Figures and Tables

**Table 1:** Elements of the quality target product profile (QTPP), their targets and justification.

QTPP Element	Target	Justification
Indication	Neuroinflammation	NDs, such as Alzheimer’s Disease and Multiple Sclerosis usually pairs with inflammation in the CNS resulting in decreasing quality of life (QoL)
Target patients	Adult (>18 years)	The listed NDs usually concern adult, mostly elderly patients.
Administration route	Nasal	The API can directly reach the CNS bypassing the first-pass effect via “nose-to-brain” route [8].
Site of activity	CNS	COX enzymes play a large role locally in the CNS mediating neuroinflammation [1].
Absorption feature	Rapid uptake by the nasal mucosa	This QTPP is closely related to dissolution and permeability, which two main factors must be fitted to the requirements of the nasal administration route. With high absorption in a short period of time a rapid onset of action can be achieved which feature can be utilized as a reliever for neuroinflammation besides sustaining therapy.
Dissolution profile	Immediate drug-release	The duration of stay is around 10–15 min due to mucociliary clearance therefore it is crucial to efficacy.
Nanocarrier	Polymeric micelles with particle size between 80 and 120 nm with monodisperse distribution	The proper particle characteristics are a critical parameter in absorption from the nasal mucosa exploiting the “nose-to-brain” pathway. Furthermore, increase in specific surface area and decrease in size contributes to higher water solubility, leading to faster dissolution.

**Table 2:** Composition and responses of the Box–Behnken factorial design.

Standard Run.	Independent Variables			Z-Average * (nm)	PdI *
	Soluplus^®^ (mg/mL)	Ethanol (mL)	1-M NaOH (mL)		
1	6.0	5.0	4.5	194.5 ± 1.8	0.411 ± 0.02
2	12.0	5.0	4.5	188.0 ± 3.9	0.299 ± 0.09
3	6.0	10.0	4.5	94.87 ± 1.1	0.155 ± 0.02
4	12.0	10.0	4.5	270.1 ± 2.0	0.513 ± 0.07
5	6.0	7.5	3.0	105.6 ± 0.9	0.164 ± 0.01
6	12.0	7.5	3.0	170.4 ± 5.6	0.477 ± 0.04
7	6.0	7.5	6.0	116.2 ± 1.5	0.140 ± 0.03
8	12.0	7.5	6.0	200.12 ± 7.3	0.344 ± 0.11
9	9.0	5.0	3.0	195.0 ± 2.3	0.377 ± 0.05
10	9.0	10.0	3.0	111.6 ± 3.0	0.114 ± 0.06
11	9.0	5.0	6.0	65.4 ± 1.6	0.401 ± 0.17
12	9.0	10.0	6.0	99.5 ± 1.7	0.444 ± 0.06
13	9.0	7.5	4.5	99.86 ± 0.5	0.134 ± 0.04
14	9.0	7.5	4.5	100.12 ± 0.7	0.155 ± 0.08
15	9.0	7.5	4.5	107.7 ± 3.1	0.163 ± 0.05

* Data are means ± SD (*n* = 3 independent formulations).

**Table 3:** Solubility, encapsulation efficiency and calculated parameters of drug-loaded polymeric micelles.

pH	S_w_ (mg/L)	S_tot_ (mg/L)	χ	P	P_M_	ΔG_s_^0^ (kJ/mol)	EE (%)
5.6	6.92 ± 0.19	5419.7 ± 1.284	0.54	782.20	77.68	−10.983	89.4
7.03	6.99 ± 0.45	5527.0 ± 1.197	0.55	789.70	78.34	−10.813	92.1
7.4	7.09 ± 0.33	5775.9 ± 0.798	0.57	825.45	81.98	−11.363	89.9

**Table 4:** Wetting properties of components and polymeric micelles.

Samples.	Θ_water_ (°)	Θ_diiodomethane_ (°)	γ^d^ (mN m^−1^)	γ^p^ (mN m^−1^)	γ (mN m^−1^)	Polarity (%)
MEL	74.1 ± 5.2	15.9 ± 3.3	44.7	9.77	54.47	17.9
Soluplus^®^	33.4 ± 0.3	16.4 ± 0.0	44.02	29.20	73.22	39.6
Physical mixture	34.3 ± 1.7	19.2 ± 2.1	43.34	29.00	72.34	40.0
Polymeric micelle	11.3 ± 0.5	23.4 ± 0.1	42.08	37.13	79.21	47.0

**Table 5:** Thermal behavior of components and the formulation.

Material	Starting Point of Weight Loss (°C)	Maximal Weight Loss at 275 °C (%)
Meloxicam	257	2.92
Soluplus^®^	241	5.33
Physical mixture	185	7.14
Polymeric micelle	103	4.98

**Table 6:** Results of DLS measurements after 12 months of physical-stability examination.

Sampling (Month)	Z-Average * (nm)	PdI *
0	100.47 ± 0.87	0.149 ± 0.01
1	100.79 ± 0.51	0.151 ± 0.04
2	100.88 ± 0.82	0.152 ± 0.02
3	100.85 ± 1.03	0.152 ± 0.03
4	101.05 ± 0.41	0.153 ± 0.05
5	101.11 ± 0.93	0.155 ± 0.01
6	101.19 ± 0.48	0.154 ± 0.04
7	101.22 ± 0.21	0.154 ± 0.06
8	101.79 ± 0.81	0.156 ± 0.07
9	101.76 ± 1.15	0.160 ± 0.04
10	101.94 ± 0.77	0.161 ± 0.03
11	102.56 ± 0.42	0.163 ± 0.08
12	103.78 ± 0.36	0.165 ± 0.04
absolute change	+3.31	+0.016

* Data are means ± SD (*n* = 3 independent formulations).

**Table 7:** Concentration of residual organic solvent in the optimized formulation.

Ethanol (mM)	Ethanol (ppm)	Maximum Residual Level * (ppm)
0.0022 ± 0.0001	0.101345 ± 0.0046	5000

* based on the ICH Q3C (R5).

**Table 8:** Physicochemical parameters of the nasal formulation.

Parameter	Value
Reconstitution time (s)	2
pH	6.49
Z-average (nm)	100.47 ± 0.87
PdI	0.149 ± 0.01
Zeta potential (mV)	−26.7 ± 0.6
Osmolality (mOsmol/L)	240
Viscosity (35 °C) (mPas)	32.5 ± 0.28

**Table 9:** Statistical analysis of the in vitro dissolution test.

Samples	%DE_10 min._	%DE_15 min._	%DE_30 min._	%DE_60 min._	RD_15 min._	MDT
MEL	2.96	3.53	4.52	5.52	1.00	11.50
Pol_mic1	72.33	78.74	86.28	91.44	22.23	3.44
Pol_mic2	73.05	79.03	87.52	93.08	22,39	3.76
Pol_mic3	73.20	79.63	87.22	91.96	22.56	3.58
Average (formulations)	72.86	79.04	87.01	92.16	22.39	3.59
SD (formulations)	0.465	0.454	0.647	0.838	0.118	0.160

**Table 10:** Results of in vitro permeability study. Flux (J); permeability coefficient (K_p_) and relative permeability at 15 min (RP_15 min_) values of MEL-loaded polymeric micelles compared to crystalline MEL.

Samples	J (µg/cm^2^/h)	K_p_ (cm/h)	RP_15 min_
Crystalline MEL	7.67	0.005752	1.00
Pol_mic1	127.33	0.095550	5.35
Pol_mic2	110.32	0.082746	4.56
Pol_mic3	127.02	0.095277	5.80
Average (formulations)	121.56	0.091191	5.24
SD (formulations)	9.73	0.007315	0.63
